# Root canal retreatment: a retrospective investigation using regression and data mining methods for the prediction of technical quality and periapical healing

**DOI:** 10.1590/1678-7757-2020-0799

**Published:** 2021-04-19

**Authors:** Bruna SIGNOR, Luciano Costa BLOMBERG, Patrícia Maria Poli KOPPER, Paulo Affonso Nonnenmacher AUGUSTIN, Marcos Vinicius RAUBER, Guilherme Scopel RODRIGUES, Roberta Kochenborger SCARPARO

**Affiliations:** 1 Universidade Federal do Rio Grande do Sul Faculdade de Odontologia Programa de Pós-graduação em Odontologia Porto Alegre Brasil Universidade Federal do Rio Grande do Sul (UFRGS), Faculdade de Odontologia, Programa de Pós-graduação em Odontologia, Porto Alegre, Brasil.; 2 Universidade Federal de Ciências da Saúde de Porto Alegre Escola de Informática Biomédica Porto Alegre Brasil Universidade Federal de Ciências da Saúde de Porto Alegre (UCFSPA), Porto Alegre, Escola de Informática Biomédica, Porto Alegre, Brasil.

**Keywords:** Endodontics, Retreatment, Decision trees, Technical quality, Periapical healing

## Abstract

**Objectives:**

This study aimed to investigate patterns and risk factors related to the feasibility of achieving technical quality and periapical healing in root canal non-surgical retreatment, using regression and data mining methods.

**Methodology:**

This retrospective observational study included 321 consecutive patients presenting for root canal retreatment. Patients were treated by graduate students, following standard protocols. Data on medical history, diagnosis, treatment, and follow-up visits variables were collected from physical records and periapical radiographs and transferred to an electronic chart database. Basic statistics were tabulated, and univariate and multivariate analytical methods were used to identify risk factors for technical quality and periapical healing. Decision trees were generated to predict technical quality and periapical healing patterns using the J48 algorithm in the Weka software.

**Results:**

Technical outcome was satisfactory in 65.20%, and we observed periapical healing in 80.50% of the cases. Several factors were related to technical quality, including severity of root curvature and altered root canal morphology (p<0.05). Follow-up periods had a mean of 4.05 years. Periapical lesion area, tooth type, and apical resorption proved to be significantly associated with retreatment failure (p<0.05). Data mining analysis suggested that apical root resorption might prevent satisfactory technical outcomes even in teeth with straight root canals. Also, large periapical lesions and poor root filling quality in primary endodontic treatment might be related to healing failure.

**Conclusion:**

Frequent patterns and factors affecting technical outcomes of endodontic retreatment included root canal morphological features and its alterations resulting from primary endodontic treatment. Healing outcomes were mainly associated with the extent of apical periodontitis pathological damages in dental and periapical tissues. To determine treatment predictability, we suggest patterns including clinical and radiographic features of apical periodontitis and technical quality of primary endodontic treatment.

## Introduction

Root canal treatment has proven to be a predictable procedure with a high success rate.^1^ Nevertheless, failures occur in 14-16% of primary endodontic treatments,^2,3^ and retreatments account for approximately 30% of the demand for endodontists.^1^ The presence of clinical symptoms and/or maintenance/progression of periapical radiolucency^4^are evaluated for determining treatment success. Moreover, poor technical quality of previous endodontic procedures and loss of coronal sealing^5^are considered when making decisions on endodontic retreatment. In this regard, satisfactory technical outcomes are considered when well-condensed root fillings are achieved along a working length of 0 to 2 mm from the radiographic apex.^6^

Previous investigations support the preference for non-surgical retreatment over endodontic surgery and show that late failures are more prone to occur in surgically treated teeth. Simultaneously, a slower healing dynamic could explain the increased success rate over time in non-surgically retreated teeth.^7^ The significantly lower healing rate in retreatments, compared to primary endodontic treatments, has been well documented.^1,8^ Within this context, persistent microbial infection is one of the major causes of failure.^5^

Several studies radiographically assessed the technical quality of root fillings, assuming it may affect the root canal treatment outcome.^9^ However, it is necessary to identify the features and patterns that could impact achieving technical quality. This is especially relevant for non-surgical endodontic retreatment, since root canal morphology may be altered,^10^increasing technical complexity.

Interestingly, most studies that evaluate endodontic retreatment success rates include samples with broadly variable characteristics,^4,7^ and few studies address whether demographic, technical, anatomical, and pathological features interfere with retreatment predictability.^1,11^ Factors such as the presence of periapical radiolucency^8^ and the incidence of interappointment flare-ups^1^ have been associated with lower success rates of root canal retreatments, but there is no consensus on this issue.^12^ Gorni and Gagliani^10^ (2004) stated that the clinical success of a retreatment depends on whether the primary endodontic treatment promoted root canal morphological alterations. Separated instruments and root perforations are potential factors to affect the clinical and radiographic success of non-surgical retreatment.^1^ Nevertheless, other authors have shown that separated instruments do not affect the periapical repair.^13^

Individualized strategies for diagnostic purposes and case selection are still required. In this regard, alternative methods for data analysis should be considered to improve technical and healing outcome prediction and thus guide clinical decisions. To date, descriptive statistics and/or logistic regression are the most widely used methods for observational studies in endodontics.^1,8^ On the other hand, advances in the field of computer sciences allow improving the ability to record and intelligently analyze large volumes of information using other approaches.^14^ Complementary approaches to purely statistical descriptive and predictive studies — including data mining strategies — are still poorly used in the dentistry field,^15^but they may significantly contribute towards knowledge discovery.^14^ Knowledge Discovery in Database (KDD) is a widely employed method and has proven to be a great resource for the identification of valid, new, understandable, and potentially useful patterns for several areas of knowledge.^14-18^

This study aimed to improve endodontic retreatment case selection by identifying frequent factors and patterns related to technical complexity and periapical healing. Therefore, we used a predictive data mining functionality to complement descriptive and regression analyses.

## Methodology

### Study design, ethical issues, and population

**Figure 1:**
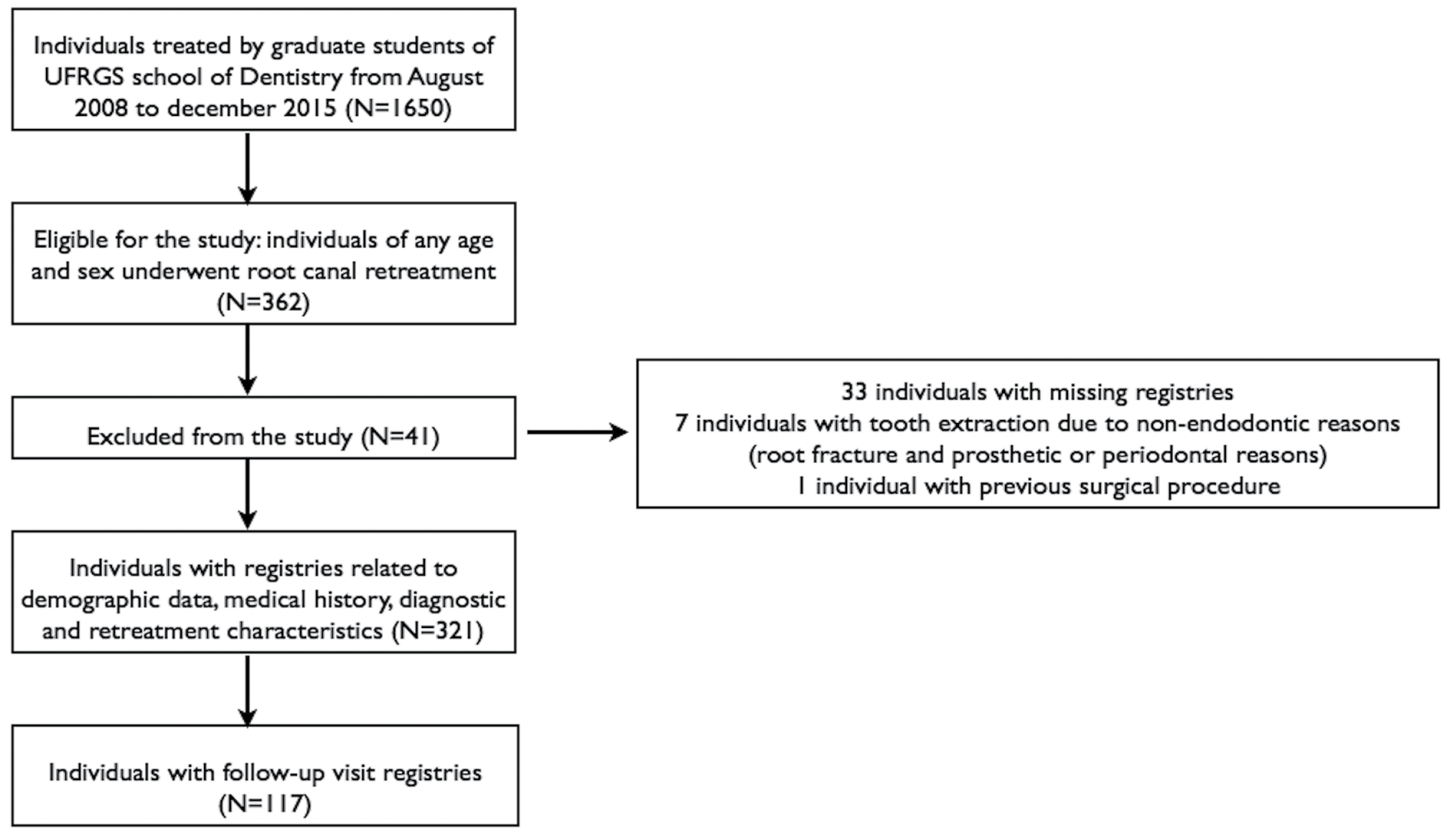
Flow chart of the study sample data

This retrospective observational study was approved by the local Ethics Committee (#2.004.117) and followed the STROBE guidelines.^19^ All consecutive patients (one tooth per patient) presenting for non-surgical root canal retreatment in the specialization program in Endodontics at the UFRGS School of Dentistry (Porto Alegre, Brazil) from August 2008 to December 2015 were included. Patients with teeth subjected to previous surgical procedures, with immature root development and/or extracted for non-endodontic reasons were excluded from the analysis. A total of 1650 teeth underwent endodontic treatment. Out of those, 362 underwent non-surgical endodontic retreatment and were eligible for the study. Finally, 321 met the inclusion criteria for assessing the technical outcomes, and 117 had follow-up records and were considered for the evaluation of healing outcomes (Figure 1).

All teeth were treated following the UFRGS specialization program in Endodontics standard protocols. In brief, it is recommended to determine the working length using apex locators. Foraminal patency should be achieved by cervical apical root canal preparation within 1 mm from the root apex. EDTA and 2% sodium hypochlorite are used as chemical auxiliaries of root canal preparation, and intracanal medication with calcium hydroxide is maintained for two weeks prior to root canal filling.

### Data collection and preprocessing

A structured electronic chart database (ECD) containing models that comprised all clinical data was developed using PHP programming language, supported by a database model created in a MySQL database management system (DBMS). Information obtained from physical records and radiographs was transferred to the web application. A total of 239 variables related to endodontic diagnosis, retreatment procedures, and follow-up visits were collected. Unnecessary features (patient identity code, date of appointments) and variables with all missing values were eliminated. Some attributes were integrated, recoded, or calculated to construct new variables.^14,16^

### Variables included in the study

#### Medical history and diagnosis

All medical variables were assessed by self-report. After data preprocessing, six were selected, including age and sex. Cardiovascular disease, hypertension, diabetes, and smoking habit were either present or absent.

Data collected during dental history taking and clinical and radiographic examination comprised 19 variables. Tooth number was recoded for obtaining tooth type and tooth location. Variables related to any clinical sign and/or symptom of periapical disease were integrated and considered either present or absent. Root resorption, canal deviation, root perforations, separated instrument, and extruded filling material (present/absent) were selected, as well as their location. Some variables were integrated to indicate whether one or more procedural accidents were present or absent. The level of the root filling and the root filling quality were classified as previously described.^8,20^ Root canal morphology (RCM) was regarded as altered if the primary endodontic treatment presented a short root filling level (>2 mm from root apex) and/or canal deviation, root perforation, and/or separated instrument. RCM was respected when these features were not observed.

All radiographs were measured by the same calibrated examiner, using the area determination tool of Image J software (National Institutes of Health, Bethesda, Maryland, USA). Intra-examiner reproducibility was tested by carrying out duplicate measurements in ten radiographs at two different time points, 20 days apart. Root canal curvature (RC) was classified according to the previously suggested stratification^21^ (Kappa index = 0.70). Periapical status (PS) was assessed based on both periapical index (PAI) scores (Kappa index = 0.80)^22^ and periapical lesion area (PLA) (ICC = 0.80). Multirooted teeth were classified according to the most severe RC, to the highest PAI score and the highest apical radiolucency.

## Endodontic retreatment

Data on endodontic retreatment comprised five variables after data preprocessing. The variables related to the occurrence of new procedural accidents (yes/no) and level after root canal retreatment^8^ were selected. The number of appointments was recoded (single/multiple appointments). The variables related to the management of procedural accidents were integrated. Satisfactory outcomes were considered when separated instruments were removed or bypassed, canal deviation was bypassed and/or root perforation was sealed, and the original root canal path was accessed, enabling proper canal instrumentation and filling. When the outcomes described were not achieved, the outcome was classified as unsatisfactory.

Radiographs taken after endodontic retreatment were analyzed to determine its technical quality (Kappa =0.78). The classification suggested by the European Society of Endodontology^6^ (2006) was adapted. Endodontic retreatment was technically satisfactory when well-condensed root fillings were achieved along a working length of 0 to 2 mm from the radiographic apex. If procedural accidents were present, the criteria for assessment of their management were applied. When satisfactory management of procedural accidents was not achieved, the technical quality of endodontic retreatment was deemed unsatisfactory. Retreatment performed on multirooted teeth with at least one root canal that did not meet the criteria for satisfactory technical quality was deemed technically unsatisfactory.

## Follow-up visits

Four variables were considered, including coronal sealing (present/absent) and type of dental restoration (definitive/temporary/absent). The follow-up period (in years) was calculated. All clinical and radiographic variables related to signs and symptoms were integrated to classify periapical healing as healed (absence of clinical signs/symptoms and absent or reduced periapical radiolucency) or failure to heal (presence of clinical signs/symptoms and unaltered or increased periapical radiolucency) (Kappa=0.83). Teeth extracted for endodontic reasons were also classified as failure to heal.

## Basic Statistical Description of Data

The final dataset contained 32 independent variables and two dependent variables, comprising the technical quality of root canal retreatment and periapical healing. To evaluate periapical healing, the technical quality of endodontic retreatment was also considered an independent variable. The frequency of missing values, the distribution of categorical variables, and the mean and standard deviation of numerical variables were calculated.

## Regression Method

Statistical tests were performed using SPSS software version 15.2 (SPSS Inc., Chicago, IL, USA). The significance level was set at 0.05. To assess factors associated with the technical quality of endodontic retreatment, all diagnosis and retreatment variables were considered, except the ones related to PS and the ones employed to classify this main outcome. To determine factors associated with periapical healing, all variables in the data set were analyzed, and the technical quality of endodontic retreatment was considered an independent variable.

Univariate associations between the selected independent variables and the dependent variables were analyzed statistically either by Fisher’s exact test or Student’s t-test. Forward stepwise logistic regression evaluated joint associations among various factors and the technical quality of endodontic retreatment. The variable ‘sign/symptom’ and all variables related to the location of procedural accidents showed a high number of missing values and were not analyzed by multiple logistic regression. Similarly, data distribution and missing data did not allow reliable multivariate models to be carried out to estimate the predictors of periapical healing.

## Data Mining Predictive Decision Trees

A .csv file containing the dataset was opened in the Waikato Environment of Knowledge Analysis (Weka - version 3.7) software, and a new .arff file was later generated to be modeled using the Weka software (www.cs.waikato.ac.nz/ml/weka). J48 classification algorithm was used.^23^ For predicting technical quality, a first experiment considered all variables related to medical history and diagnosis. In a second experiment, only variables related to diagnosis were kept, and technical errors of the previous endodontic treatment were grouped into the RCM variable. One experiment was performed for periapical healing prediction, considering all potential risk factors in the data set. For the three experiments, the minimum number of instances per leaf node was set to 7. The accuracy and stability of induced decision trees were provided by Weka and tested using the cross-validation procedures.

## Results

### Data Distribution

The distribution of prognostic factors for endodontic retreatment related to medical history, diagnosis, endodontic retreatment, and follow-up visits is summarized in Table 1. Molars accounted for the most frequent tooth type (42.67%). Procedural accidents were present in 12.14% of the cases. Root fillings were regarded as unsatisfactory in 73.52% of individuals. Root resorption was observed in 16 teeth, and all of them were located in the apical third of the root. New procedural accidents were not observed. After endodontic retreatment, the technical outcome was satisfactory in 65.20%, and 53.84% of preexisting procedural accidents had a satisfactory technical outcome. Follow-up periods had a mean of 4.05 years. We observed healing in 80.50% of the cases. Coronal sealing was present in 93.69% of individuals.

**Table 1:** Distribution of variables related to medical history, diagnosis, endodontic retreatment, and follow-up visits (N = 321)

Variables related to medical history and diagnosis		n (%)	Mean (sd)
Age (309)	Young (≤19 years)	5 (1.61%)	
	Adult (20-59 years)	231 (74.75%)	-
	Elderly (≥60 years)	73 (23.62%)	
	Missing values	12	
Sex (318)	Man	84 (26.47%)	
	Woman	234 (75.52%)	-
	Missing values	3	
Cardiovascular disease (306)	Absent	284 (89.87%)	
(medical diagnosis of angina, coronary artery disease, stroke, and/or myocardial infarction)	Present	22 (6.96%)	
	Missing values	15	-
Hypertension (306)	Absent	259 (84.64%)	
(systolic blood pressure ≥ 140 mmHg and diastolic blood pressure ≥ 90 mmHg)	Present	47 (15.35%)	
	Missing values	15	-
Diabetes (306)	Absent	295 (96.40%)	
(history of blood glucose tests ≥ 150 mg/dL)	Present	11 (3.59%)	-
	Missing values	15	
Smoking habit (306)	Absent	277 (90.52%)	
(current smoker)	Present	29 (9.47%)	-
	Missing values	15	
Sign/symptom (245)	Absent	77 (31.42%)	
	Present	168 (68.57%)	-
	Missing values	76	
Tooth type (321)	Anterior	95 (29.59%)	
	Pre-molar	89 (27.72%)	-
	Molar	137 (42.67%)	
Tooth location (321)	Maxilla	191 (59.50%)	
	Mandible	130 (40.49%)	-
Level root filling (321)	> 2 mm	220 (68.53%)	
	0-2 mm	92 (28.66%)	-
	Beyond the apex	9 (2.80%)	
Root filling quality (321)	Satisfactory	85 (26.47%)	
	Unsatisfactory	236 (73.52%)	-
Root curvature (321)	Straight (≤ 5°)	173 (53.89%)	
	Moderate (10 – 20°)	115 (35.82%)	-
	Severe (≥ 25 – 70°)	33 (10.28%)	
Periapical status (PAI) (321)	1	70 (21.80%)	
	2	47 (14.64%)	
	3	2 (0.62%)	-
	4	103 (32.08%)	
	5	99 (30.84%)	
Periapical lesion area (321)	-	-	5.37±15.08
Root resorption (321)	Absent	305 (95.01%)	
	Present	16 (4.98%)	-
Root resorption location (16)	Cervical	0	
	Middle	0	-
	Apical	16 (100%)	
Canal deviation (321)	Absent	309 (96.26%)	
	Present	12 (3.73%)	-
Canal deviation location (12)	Coronal	2 (16.66%)	
	Middle	3 (25%)	-
	Apical	7 (58.33%)	
Root perforations (321)	Absent	310 (96.57%)	
	Present	11 (3.42%)	-
Root perforations location (11)	Furcation area	3 (27.27%)	
	Coronal	4 (36.36%)	
	Middle	3 (27.27%)	-
	Apical	1 (9.09%)	
Separated instruments (321)	Absent	308 (95.95%)	
	Present	13 (4.04%)	-
Separated instruments location (13)	Coronal	2 (15.38%)	
	Middle	6 (46.15%)	-
	Apical	5 (38.46%)	
Extruded filling material (321)	Absent	313 (97.50%)	
	Present	8 (2.48%)	-
Procedural accidents (321)	Absent	282 (87.85%)	
	Present	39 (12.14%)	-
Root canal morphology (321)	Respected	291 (90.65%)	
	Altered	30 (9.34%)	-
Variables related to endodontic retreatment		n (%)	
Level of root filling after retreatment (321)	> 2 mm	51 (15.88%)	
	0-2 mm	211 (65.73%)	-
	Beyond the apex	59 (18.38%)	
Number of appointments (321)	Multiple Appointments	302 (94.08%)	
	Single Appointments	19 (5.91%)	-
Technical outcome of accident management (39)	Satisfactory	21 (53.84%)	
	Unsatisfactory	18 (46.15%)	-
Technical quality of endodontic retreatment (319)	Satisfactory	208 (65.20%)	
	Unsatisfactory	111 (34.79%)	-
	Missing values	2	
Variables related to follow-up visits		n (%)	
Coronal sealing (111)	Absent	7 (6.30%)	
	Present	104 (93.69%)	-
	Missing values	6	
Type of dental restoration (111)	Definitive	95 (85.58%)	
	Temporary	12 (10.81%)	
	Absent	4 (3.60%)	-
	Missing values	6	
Follow-up period (years) (117)	-	-	4.05±1.42
Periapical healing (117)	Healed	95 (80.50%)	
	Failure	22 (19.49%)	-

### Regression Method Results

Univariate analysis revealed that tooth type, RC, procedural accidents, canal deviation location, extruded filling material, and RCM were significantly associated with the technical quality of endodontic retreatment (p<0.05) (Table 2). The stepwise regression model identified RC and RCM as being significantly predictive of the technical quality of root canal retreatment outcome (p<0.05), while extruded filling material presented a borderline nonsignificant association with this main outcome (Table 3).

**Table 2:** Prognostic factors associated with the technical quality of endodontic retreatment (N = 321)

Variables		n	Satisfactory technical quality of endodontic retreatment - n(%)	p value
Sign/symptom (245)	Absent	77	48 (62.3%)	0.566 ▲
	Present	168	112 (66.3%)	
	Missing values	76	-	
Tooth type (321)	Anterior	95	71 (74.7%)	0.001▲*
	Pre-molar	89	65 (73%)	
	Molar	137	72 (53.3%)	
Tooth location (321)	Maxilla	191	131 (68.6%)	0.150□
	Mandible	130	77 (60.2%)	
Root curvature (321)	Straight	173	128 (74%)	<0.001▲*
	Moderate	115	67 (58.8%)	
	Severe	33	13 (40.6%)	
Root resorption location (321)	Absent	305	200 (66%)	0.280▲
	Apical third	16	8 (50%)	
Canal deviation (321)	Absent	309	203 (66.1%)	0.119□
	Present	12	5 (41.7%)	
Canal deviation location (12)	Coronal	2	2 (100%)	<0.001▲*
	Middle	3	3 (100%)	
	Apical	7	0 (0)	
Root perforations (321)	Absent	310	202 (65.4%)	0.743▲
	Present	11	6 (60%)	
Root perforations location (11)	Furcation area	3	1 (33.3%)	0.121□
	Coronal	4	2 (66.7%)	
	Middle	3	3 (100%)	
	Apical	1	0 (0)	
Separated instruments (321)	Absent	308	201 (65.7%)	0.386▲
	Present	13	7 (53.8%)	
Separated instruments location (13)	Coronal	2	2 (100%)	0.235▲
	Middle	6	3 (50%)	
	Apical	5	2 (40%)	
Extruded filling material (321)	Absent	313	206 (66.2%)	0.005▲*
	Present	8	2 (66.7%)	
Procedural accidents (321)	Absent	282	192 (68.3%)	0.002▲*
	Present	39	16 (42.1%)	
Root canal morphology (321)	Respected	291	197 (67.9%)	0.002▲*
	Altered	30	11 (37.9%)	
Number of appointments (321)	Multiple Appointments	302	193 (64.3%)	0.225▲
	Single Appointment	19	15 (78.9%)	

* statistical significance; ▲ Fisher´s exact test; □ Students’ t test

**Table 3:** Stepwise forward logistic regression analysis for technical quality of non-surgical root canal retreatment (N = 321)

Prognostic factor	OR	95% CI	p Value
Root curvature	.480	.336 - .686	<.0001
Root Canal Morphology	.401	.169 - .950	.038
Extruded filling material	.302	.086 - 1.055	.061

Univariate analysis showed that tooth group, root resorption, and PLA were significantly associated with periapical healing (p<0.005). In contrast, PAI, age range, root filling level after retreatment, and technical quality of endodontic retreatment showed a borderline nonsignificant association with periapical healing (Table 4).

**Table 4:** Prognostic factors associated with periapical healing in endodontic retreatment (N = 117)

Variables		n	Healed (n/%)	p Value
Age (114)	Young	1	1 (100%)	0.079□
	Adult	84	64 (76.2%)	
	Elderly	29	27 (93.1%)	
	Missing values	3	-	
Sex (116)	Man	34	26 (76.5%)	0.610▲
	Woman	82	67 (81.7%)	
	Missing value	1		
Systemic chronic condition (110)	Absent	47	39 (83%)	0.631▲
	Present	63	49 (77.8%)	
	Missing values	7	-	
Sign/symptom (84)	Absent	21	15 (71.4%)	0.370▲
	Present	63	51 (81%)	
	Missing values	33	-	
Tooth type (117)	Anterior	33	25 (72.7%)	0.015▲*
	Pre-molar	37	35 (94.6%)	
	Molar	47	35 (74.5%)	
Tooth location (117)	Maxilla	69	56 (81.2%)	0.816▲
	Mandible	48	38 (79.2%)	
Level root filling (117)	> 2 mm	34	28 (82.4%)	0.916▲
	0-2 mm	79	63 (79.7%)	
	Beyond the Apex	4	3 (75%)	
Root filling quality (117)	Satisfactory	40	31 (77.5%)	0.627▲
	Unsatisfactory	77	63 (81.8%)	
Root curvature (117)	Straight	67	53 (79.1%)	0.552▲
	Moderate	35	30 (85.7%)	
	Severe	15	11 (73.3%)	
Periapical status (PAI) (117)	1	16	14 (87.5%)	0.075▲
	2	16	14 (87.5%)	
	3	-	-	
	4	45	39 (87.6%)	
	5	40	27 (67.5%)	
Periapical lesion area		117	-	0.018■*
Root resorption (117)	Absent	113	93 (82.3%)	0.024▲*
	Present	4	1 (25%)	
Canal deviation (117)	Absent	111	90 (81.1%)	0.336▲
	Present	6	4 (66.7%)	
Canal deviation location (6)	Coronal	1	0 (0)	0.208▲
	Middle	1	1 (100%)	
	Apical	4	3 (75%)	
Root perforations (117)	Absent	115	93 (80.9%)	0.356▲
	Present	2	1 (50%)	
Separated instruments (117)	Absent	111	90 (81.1%)	0.336▲
	Present	6	4 (66.7%)	
Separated instruments location (6)	Coronal	1	0 (0)	0.148▲
	Middle	3	2 (66.7%)	
	Apical	2	2 (100%)	
Extruded filling material (116)	Absent	112	91 (81.3%)	0.101▲
	Gutta-percha	1	0 (0)	
	Endodontic sealer	3	3 (100%)	
	Both	-	-	
	Missing	1		
Procedural accidents (117)	Absent	100	82 (82%)	0.322▲
	Present	17	12 (70.6%)	
Root canal morphology (117)	Respected	105	86 (81.9%)	0.249▲
	Altered	12	8 (66.7%)	
Level root filling after retreatment (117)	> 2 mm	72	62 (86.1%)	
	0-2 mm	23	15 (65.2%)	0.098▲
	Beyond the apex	22	17 (77.3%)	
Number of appointments (117)	Multiple appointments	108	87 (80.6%)	0.999▲
	Single appointment	9	7 (77.8%)	
Technical outcome of accident management (17)	No	9	7 (77.8%)	0.620▲
	yes	8	5 (62.5%)	
Type of dental restoration (110)	Definitive	94	80 (85.1%)	0.691▲
	Temporary	12	11 (91.7%)	
	Absent	4	3 (75%)	
	Missing values	7	-	
Follow-up period (years) (117)		117	-	0.444□
Technical quality of endodontic retreatment (115)	Satisfactory	71	62 (87.3%)	0.080▲
	Unsatisfactory	44	32 (72.7%)	
	Missing values	2		

### Data mining results

**Figure 2:**
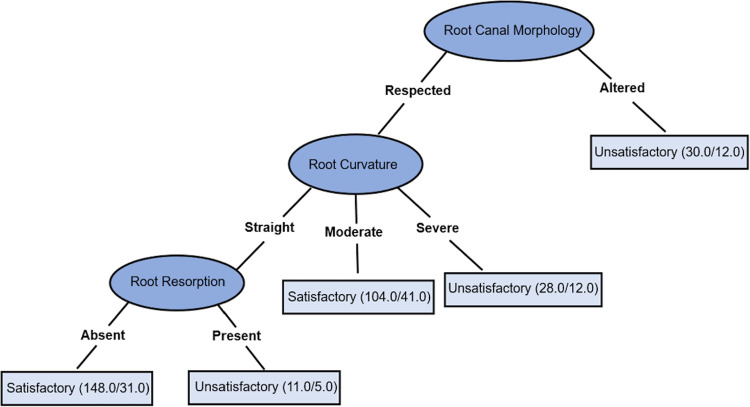
Decision tree A: Prediction of technical quality of non-surgical root canal retreatment, including variables related to diagnosis - (1) For canal deviation located in the apical third of the root, the technical quality of endodontic retreatment was unsatisfactory; (2) For absent canal deviation, severe RC, and tooth located in the mandible, the technical quality was unsatisfactory, whereas, for tooth located in the maxilla, the technical quality was satisfactory; (3) For moderate RC and separated instrument, the technical quality was unsatisfactory; (4) A straight RC immediately predicted that technical quality was satisfactory. The values in parentheses point out the total number of correctly/incorrectly classified instances in each leaf per node (Accuracy of 62.38%)

Three decision trees were created. The decision tree shown in Figure 2 (tree A) can be read as follows: (1) For canal deviation located in the apical third of the root, the technical quality of endodontic retreatment was unsatisfactory; (2) For absent canal deviation, severe RC, and tooth located in the mandible, the technical quality was unsatisfactory, whereas, for tooth located in the maxilla, the technical quality was satisfactory; (3) For moderate RC and separated instrument, the technical quality was unsatisfactory; (4) A straight RC immediately predicted that technical quality was satisfactory.

**Figure 3:**
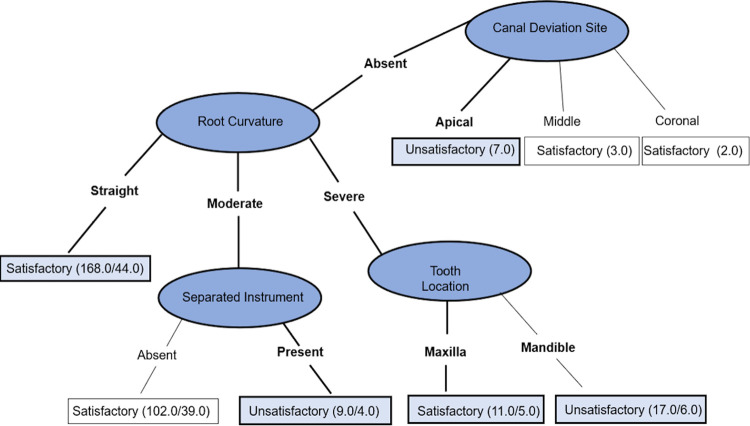
Decision tree B: Prediction of technical quality of non-surgical root canal retreatment, including variables related to demographic data, medical history, and diagnosis - (1) For altered RCM, the technical quality was unsatisfactory; (2) For respected RCM and severe RC, the technical quality was unsatisfactory, whereas, for moderate RC, it was satisfactory; (3) For respected RCM, straight RC, and presence of root resorption, the technical quality was unsatisfactory. When root resorption was absent, the technical quality was satisfactory. The values in parentheses point out the total number of correctly/incorrectly classified instances in each leaf per node (Accuracy of 66.66%)

After grouping RCM and excluding the variables related to demographic and medical data, the decision tree (Figure 3 – tree B) can be read as follows: (1) For altered RCM, the technical quality was unsatisfactory; (2) For respected RCM and severe RC, the technical quality was unsatisfactory, whereas, for moderate RC, it was satisfactory; (3) For respected RCM, straight RC, and presence of root resorption, the technical quality was unsatisfactory. When root resorption was absent, the technical quality was satisfactory.

**Figure 4:**
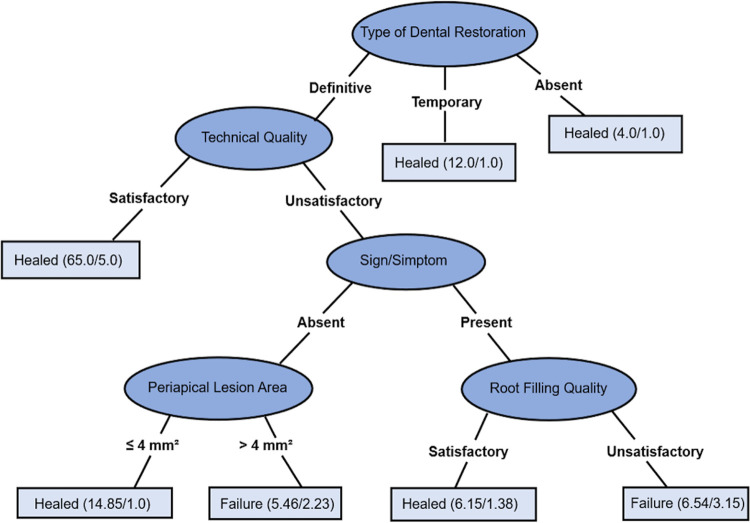
Decision tree C: Prediction of periapical healing of non-surgical root canal retreatment, including variables related to demographic data, medical history, diagnosis, endodontic retreatment characteristics, and follow-up data – (1) For absent coronal sealing or presence of temporary dental restoration at the time of follow-up visit, periapical healing was classified as healed. (2). For definitive dental restoration and satisfactory technical quality of endodontic retreatment, periapical healing was classified as healed. (3) For definitive dental restoration at the follow-up visit, unsatisfactory technical quality of endodontic retreatment, presence of signs/symptoms at the time of diagnosis, and unsatisfactory root fillings in the primary endodontic treatment, periapical healing was classified as failure to heal; but for satisfactory root filling quality of the primary endodontic treatment, periapical healing was classified as healed; (4) For identification of definitive dental restoration at the follow-up visit, unsatisfactory technical quality of endodontic retreatment, absent signs/symptoms, and periapical lesion area greater than 4 mm2 at the time of diagnosis, periapical healing was classified as failure to heal. However, for periapical lesion areas smaller than 4 mm2, periapical healing was classified as healed. The values in parentheses indicate the total number of correctly/incorrectly classified instances in each leaf per node (Accuracy of 79.66%)

Regarding the periapical healing binary class, the J48 classifier generated one decision tree (Figure 4 – tree C) that can be read as follows: (1) For absent coronal sealing or presence of temporary dental restoration at the time of follow-up visit, periapical healing was classified as healed. (2). For definitive dental restoration and satisfactory technical quality of endodontic retreatment, periapical healing was classified as healed. (3) For definitive dental restoration at the follow-up visit, unsatisfactory technical quality of endodontic retreatment, presence of signs/symptoms at the time of diagnosis, and unsatisfactory root fillings in the primary endodontic treatment, periapical healing was classified as failure to heal. However, for satisfactory root filling quality of the primary endodontic treatment, periapical healing was classified as healed; (4) For identification of definitive dental restoration at the follow-up visit, unsatisfactory technical quality of endodontic retreatment, absent signs/symptoms, and periapical lesion area greater than 4 mm^2^ at the time of diagnosis, periapical healing was classified as failure to heal. However, for a periapical lesion area smaller than 4 mm^2^, periapical healing was classified as healed.

## Discussion

This investigation analyzed frequent patterns and factors related to technical quality and periapical healing in endodontic retreatment.

Satisfactory technical quality was achieved in 65.20% of the current study sample. A meta-analysis evaluating primary endodontic treatment performed by undergraduate students showed 48% of acceptable technical outcomes.^9^ Operators’ level of experience (graduate students) and the use of technologies, such as nickel-titanium files, electronic apex locator, and operative microscope, can be related to these differences. Accordingly, a previous study showed that technologies improve the technical standards of endodontic treatment.^24^ Periapical healing was observed in 80.50% of the follow-up patients. This outcome is similar to the one observed in other studies on endodontic retreatments performed by graduate students^3^ and by one specialist.^1^

We observed that frequent patterns and factors affecting technical outcomes of endodontic retreatment included root canal morphological features and its alterations resulting from primary endodontic treatment. Healing outcomes were mainly influenced by the extent of apical periodontitis pathological damages in dental and periapical tissues. Presence of symptoms and technical quality of primary endodontic treatment should also be considered to determine treatment predictability.

Radiographic evaluation of technical and healing outcomes of endodontic treatment through periapical radiographs is less sensitive than cone-beam computed tomography (CBCT),^25-29^ which can be considered a limitation of this study. However, due to ethical reasons and considering the observational design of this study, the standard protocols used for diagnosis and follow-up were not altered, and we based the data collection on clinical and radiographic records. To minimize this limitation, we assessed periapical status for a mean follow-up period of 4.05 years and considered clinical information to determine healing or failure. Moreover, besides PAI, we used differences in the periapical lesion area to assess healing outcomes. Another limitation is that we obtained medical history data using self-reports, which may underreport some general health variables.^30^ Thus, the absence of association between systemic conditions and periapical healing must be interpreted with caution.

For the first time, KDD was used to predict technical and healing outcomes of endodontic retreatment. KDD included selection, preprocessing, and translation of raw data into relevant information.^17^ Data mining is an essential step of KDD in which intelligent methods are applied to extract data patterns.^14^ The induction of decision trees was preferred among several data mining functionalities because this analysis can handle multidimensional data and provides a visual and analytical decision support tool.^18^ Recent studies used these algorithms to identify patterns related to periodontal disease^14^ and to assess the association between endodontic pathologies and cardiovascular diseases.^15^ J48 was the algorithm used for the analysis performed herein. It defines the possible decision tree employing a hill-climbing search based on the statistical property measure called information gain. An advantage of such a method is that it automatically handles nonlinearity and interactions.^23^ Instead of predicting risk factors, decision trees provide the frequent patterns associated with an outcome.^23^ In this study, decision trees provided additional insights that may help improve endodontic retreatment case selection. Since this method may omit the analysis of important predictors, and a reduced number of samples that fit into some of the subclasses may influence the trees’ accuracy, it was complemented by descriptive and regression analysis.^16^

While altered RCM was associated with unsatisfactory technical quality by regression analyses, decision tree B showed that severe RC predicted the unsatisfactory technical quality of endodontic retreatment, even in those cases in which RCM was respected in the primary endodontic treatment. Accordingly, a previous investigation demonstrated that root curvatures might hamper working length accessibility, affecting the incidence of procedural errors.^31^ In agreement, a significant association between tooth type and technical quality of endodontic retreatment was observed, which may be related to anatomical complexity and high frequency of moderate and severe RC in molars.^24^

Although the association of root resorption with technical quality was not assessed by regression methods, decision tree B suggested that unsatisfactory technical quality of endodontic retreatment is affected in straight root canals, without RCM alterations, and with apical resorption. A possible explanation for this finding is that root resorption may alter the shape and position of the apical foramen and apical constriction, increasing the chance of over instrumentation and subsequent overfilling of the root canal.^32^ Data mining also indicated that the technical quality in root canals with severe RC was easier to be achieved in teeth located in the maxilla, in agreement with a previous study on primary root canal treatment.^20^ Notwithstanding, radiographic technical quality might be overestimated, especially in maxillary teeth because of the frequent superimposition of anatomical structures.^26,28^

We found a significant association between the overall incidence of procedural accidents and unsatisfactory root canal retreatment. Additionally, decision tree A identified that unsatisfactory technical outcomes are common in the presence of separated instruments, moderate RC, and the absence of canal deviation. In this regard, studies show that fragments located inside or beyond the curvature are difficult to be bypassed or removed.^33^

Both data mining analysis and Fisher’s exact test showed that samples with root canal deviation in the apical third were more likely to be associated with unsatisfactory technical quality. A previous study demonstrated that the frequency of ledged root canals was significantly greater in molars than in anterior teeth.^31^ However, the study did not assess the feasibility of achieving adequate technical outcomes in root canals with deviations in different thirds of the root, as demonstrated herein.

The presence of extruded filling material in the primary endodontic treatment was associated with unsatisfactory technical quality. Previous studies did not assess the impact of overfilling prior to endodontic retreatment, but we observed that over instrumentation – which undoubtedly favors overfilling – provoked cementum perforations and/or zipping in the apical third of root canals.^34^

PLA appeared to be a more sensitive predictor of periapical healing than did PAI. The current results showed that a larger PLA is associated with failure, in line with a previous investigation.^35^ It has been suggested that larger lesions indicate cystic transformation or extra radicular infections, which would render endodontic retreatment ineffective.^12,36^ Besides, decision tree C (Figure 4) showed that small radiolucencies were likely to heal even when ideal technical outcomes were not achieved, whereas microorganism quantities and virulence were subcritical to sustain periapical inflammation.^12,36^ As previously suggested, PLA may modify the interactions between intraaticular infection and host responses.^36^ Further studies are needed to elucidate differences in the biological interactions taking place in the pathogenesis of large periapical lesions.

Technical quality showed a borderline nonsignificant association with periapical healing. Previous studies separately assessed the variables that compound the classification of technical outcomes used herein. They demonstrated that overfilling might delay healing – or even predispose to treatment failure – caused by a foreign body reaction.^8,11,12^ On the other hand, underfilling is frequently related to the inability to debride the apical segment of the root canal, which may harbor persistent intracanal infection.^36^ Moreover, retreated teeth with appropriate condensed root fillings exhibit higher success rates.^8^

Interestingly, decision tree C showed unsatisfactory root filling quality in primary endodontic treatment appeared to predispose to treatment failure. Previous studies revealed that root filling materials constitute substrate layers compatible with the physicochemical surface features of various microorganisms, thus allowing for bacterial adhesion and biofilm formation.^37^ Therefore, besides being harbored within inaccessible areas of the root canal system, microorganisms that survive root canal chemo-mechanical disinfection can attach to obturation materials, which could be favored by poor root fillings. In a study comparing the bacterial flora of standard root canal samples retrieved from obturation materials,^38^ nine species were recovered from the filling materials alone, emphasizing the microbiological importance of additional obturation material sampling in persistent infections to improve the understanding of the etiology of healing failure. Further investigations are required to evaluate whether the quality of root fillings of previous endodontic treatment affects the antimicrobial efficacy of the available protocols used for root canal disinfection in non-surgical retreatment.

At odds with previous findings,^10^ neither decision trees nor traditional statistics showed an association between endodontic retreatment failure and altered RCM. Unlike the criteria used herein, the investigation included cases with internal root resorption unsealed by former treatment in the altered RCM group and the presence of separate files and inadequate levels of filling in the respected RCM category. Moreover, the referred study^10^ observed the impact of preoperative morphological alterations on endodontic retreatment success, but it did not assess the technical quality of endodontic retreatment. Hence, other features related to technical outcomes could have affected healing.

A previous study^39^revealed that poor clinical outcomes might be expected in cases with inappropriate coronal restoration, which was not verified herein. The small number of samples with absent coronal restoration and the possibility of recent coronal sealing losses might have influenced the healing outcome. Unfortunately, the period of absent tooth restoration could not be evaluated. An *in vitro* study found that the amount of saliva penetration through root fillings should be considered clinically significant only in root canals that have been exposed to the oral cavity for at least three months.^40^

As previously demonstrated,^11,12^root resorption was associated with healing failure. Morphological alterations – combined with increased susceptibility to over instrumentation and overfilling in teeth with apical resorption^12^ – certainly play a role in preventing a satisfactory apical seal. Poor adaptation of the filling material to the irregularities of the root canal should allow tissue fluid and inflammatory exudates to percolate and continue to support the growth of microorganisms.^12^

Similar to other studies,^1,11^we observed lower success rates for endodontic retreatment performed on molars. Anatomical challenges hinder the elimination of root canal infection, which probably ends up affecting periapical healing. Unlike those studies, we observed a lower healing rate than premolars for anterior teeth (Table 4). This outcome is not in line with the technical quality of endodontic retreatment, which is probably related to the coexistence of other risk factors not assessed in the current analysis.

## Conclusion

We found that the technical quality of endodontic retreatment is associated with several risk factors, including the severity of RC and altered RCM. We should consider procedural accidents to classify case complexity since they are especially relevant in the apical third of the roots. PLA, tooth type, and apical resorption proved to be significantly associated with healing failure. Base on the decision trees, we suggest considering risk factors and patterns combining different variables to define technical complexity and periapical healing predictability. Straight root canals combined with apical root resorption might prevent satisfactory technical outcomes. Large periapical lesions and poor root filling quality in primary endodontic treatment appeared to predispose to treatment failure. Data distribution and missing data did not allow reliable multivariate models to be carried out to estimate the predictors of periapical healing. Additional future studies should assess variable operators’ experience and include larger samples to enable further analysis.
